# Microglial Activation and Antioxidant Responses Induced by the Parkinson’s Disease Protein α-Synuclein

**DOI:** 10.1007/s11481-012-9401-0

**Published:** 2012-10-10

**Authors:** Dawn Béraud, Hannah A. Hathaway, Jordan Trecki, Sergey Chasovskikh, Delinda A. Johnson, Jeffrey A. Johnson, Howard J. Federoff, Mika Shimoji, Timothy R. Mhyre, Kathleen A. Maguire-Zeiss

**Affiliations:** 1 Department of Neuroscience, Georgetown University Medical Center, NRB EP08, 3970 Reservoir Road NW, Washington, DC 20057 USA; 2 Interdisciplinary Program in Neuroscience, Georgetown University Medical Center, Washington, DC USA; 3 Department of Pharmacology, Georgetown University Medical Center, Washington, DC USA; 4 Lombardi Comprehensive Cancer Center, Georgetown University Medical Center, Washington, DC USA; 5 Division of Pharmaceutical Sciences, University of Wisconsin-Madison, Madison, WI USA; 6 Waisman Center, Division of Pharmaceutical Sciences, Molecular and Environmental Toxicology Center, University of Wisconsin-Madison, Madison, WI USA; 7 Department of Neurology, Georgetown University Medical Center, Washington, DC USA; 8 Present Address: Office of Diversion Control, Drug & Chemical Evaluation Section, Drug Enforcement Administration, 8701 Morrisette Drive, Springfield, VA 22152 USA

**Keywords:** Synucleinopathy, Microglia, Toll like receptors, Innate immunity, Nrf2, Antioxidant enzymes

## Abstract

Parkinson’s disease (PD) is the second most common age-related neurodegenerative disorder typified by tremor, rigidity, akinesia and postural instability due in part to the loss of dopamine within the nigrostriatal system. The pathologic features of this disorder include the loss of substantia nigra dopamine neurons and attendant striatal terminals, the presence of large protein-rich neuronal inclusions containing fibrillar α-synuclein and increased numbers of activated microglia. Evidence suggests that both misfolded α-synuclein and oxidative stress play an important role in the pathogenesis of sporadic PD. Here we review evidence that α-synuclein activates glia inducing inflammation and that Nrf2-directed phase-II antioxidant enzymes play an important role in PD. We also provide new evidence that the expression of antioxidant enzymes regulated in part by Nrf2 is increased in a mouse model of α-synuclein overexpression. We show that misfolded α-synuclein directly activates microglia inducing the production and release of the proinflammatory cytokine, TNF-α, and increasing antioxidant enzyme expression. Importantly, we demonstrate that the precise structure of α-synuclein is important for induction of this proinflammatory pathway. This complex α-synuclein-directed glial response highlights the importance of protein misfolding, oxidative stress and inflammation in PD and represents a potential locus for the development of novel therapeutics focused on induction of the Nrf2-directed antioxidant pathway and inhibition of protein misfolding.

## Introduction

In this invited paper we review existing literature, which supports the concept that the Parkinson’s disease (PD) protein α-synuclein plays a role in microglial activation and oxidative stress. In addition, we provide new evidence that α-synuclein overexpression induces an antioxidant response and that specific conformers of α-synuclein cause complex morphological and functional microglial responses which involve both proinflammatory molecules and phase-II antioxidant enzymes. We suggest that this multifaceted α-synuclein-directed glial response represents a nexus between protein misfolding, oxidative stress and inflammation providing a potential therapeutic locus to halt the progression of PD.

### PD and α-synuclein

PD is the most common age-related progressive neurodegenerative movement disorder affecting over 4 million people worldwide a number that is expected to double by 2030 (Dorsey et al. 2007). One important locus of this disorder is the nigrostriatal system where there is invariant loss of substantia nigra pars compacta (SNpc) dopamine neurons and striatal presynaptic terminals. A hallmark pathological feature of PD is the presence of large cytoplasmic inclusions of protein in the remaining dopamine neurons and neurites (Rodrigues e Silva et al. 2010). These so-called Lewy bodies and Lewy neurites are enriched in fibrillar α-synuclein (Spillantini et al. 1997; Baba et al. 1998; Spillantini et al. 1998). Familial forms of this disorder have been identified linking the gene that encodes α-synuclein, *SNCA,* as well as a number of other genes to PD pathogenesis (reviewed in (Lesage and Brice 2009; Hardy 2010; Martin et al. 2011)). Later it was discovered that duplication or triplication of *SNCA* causes an autosomal dominant form of PD where disease severity is linked to gene dosage (Singleton et al. 2003; Chartier-Harlin et al. 2004; Singleton et al. 2004). Importantly, genome wide association (GWA) studies implicate *SNCA* with an increased risk of developing sporadic PD, indicating that this protein plays a central role in both sporadic and familial PD (Edwards et al. 2010). Furthermore, the fact that mutations in or overexpression of *SNCA* cause PD indicates an α-synuclein toxic-gain-of-function mechanism in this neurodegenerative disorder. Although the exact mechanism by which α-synuclein leads to PD is not completely understood there is evidence that the propensity of this protein to misfold into toxic oligomers promotes disease (reviewed in (El-Agnaf and Irvine 2000; Cookson and van der Brug 2008)).

α-Synuclein is also involved in several other neurodegenerative disorders aptly named synucleinopathies (e.g., multiple system atrophy, diffuse Lewy body disease), which have disparate initiating factors, multiple affected neurotransmitter systems and different cell loci of disease (i.e., dopamine neurons, cortical neurons, oligodendrocytes) suggesting a multifaceted pathogenic mechanism. Thus far, in some in vivo and in vitro models, α-synuclein overexpression, accumulation and/or oligomer formation is accompanied by cell dysfunction or death (El-Agnaf et al. 1998; Masliah et al. 2000; Lee et al. 2001; Kirik et al. 2002; Danzer et al. 2007; Periquet et al. 2007; Desplats et al. 2009). In other overexpression models, α-synuclein does not result in robust neuronal death, which may be a function of the cell type expressing this protein and the amount of α-synuclein expressed per cell (e.g., the formation of toxic oligomers) (Matsuoka et al. 2001; Richfield et al. 2002; Colapinto et al. 2006). The formation of various α-synuclein protein conformers is promoted by molecular crowding, dopamine modification, temperature, pH, metal binding, pesticides and oxidative stress suggesting that the cellular milieu determines how this protein misfolds (Uversky et al. 2001a, c, b, d, 2002, 2007). There are a large number of mechanistic studies suggesting that α-synuclein’s toxic-gain-of-function is related to misfolding of this protein and consequent effects on mitochondria, proteasome and lysosome function (Hsu et al. 2000; Tanaka et al. 2001; Elkon et al. 2002; Meredith et al. 2002; Giasson and Lee 2003; Lindersson et al. 2004; Giorgi et al. 2006; Martin et al. 2006; Devi et al. 2008; Emmanouilidou et al. 2010b). Impairment of these key organelles can lead to α-synuclein-induced oxidative stress. In turn reactive oxygen and nitrogen species incite α-synuclein aggregation putting into motion a feed-forward cycle of synuclein-induced stress (Hashimoto et al. 1999; Giasson et al. 2000; Paxinou et al. 2001; Ischiropoulos 2003). Antioxidant compounds inhibit the formation of and destabilize preformed α-synuclein fibrils in vitro (Ono and Yamada 2006) and have some protective effects in models of PD (Trinh et al. 2008; Beal 2011; Martin et al. 2012; Martinez-Banaclocha 2012).

In the nigrostriatal system dopamine is a prominent source of reactive oxygen species as this neurotransmitter can auto-oxidize when not sequestered in vesicles. Antioxidant enzymes respond to this enhanced oxidative stress to prevent neuronal damage. However when there is an excess of free dopamine as might occur when presynaptic terminals degenerate or vesicle recycling is impaired the amount of oxidized dopamine and highly reactive dopamine quinone intermediates increases leading to neurotoxicity (Asanuma et al. 2003; Galvin 2006). Dopamine quinones are known to irreversibly modify proteins altering their normal function, for example and relevant to our discussion, dopamine oxidatively modifies α-synuclein leading to the stabilization of the toxic protofibrillar structure (i.e., oligomeric intermediates) (Conway et al. 2001). Dopamine modification of α-synuclein also has cellular consequences as dopamine-modified α-synuclein inhibits chaperone-mediated autophagy, which would render dopamine neurons more vulnerable to subsequent toxicants or stressors (Martinez-Vicente et al. 2008). Therefore the nigrostriatal system may exhibit enhanced vulnerability in PD because this region has high levels of oxidative stress and is enriched in α-synuclein as well as dopamine (Maguire-Zeiss et al. 2005; Mosharov et al. 2009; Surmeier et al. 2010).

### Microglial activation in PD

Microglia, the resident immune cells in the brain, continuously monitor and react to their microenvironment. Engagement of these cells with pathogen-associated molecular patterns (PAMPs) such as bacterial- and viral-derived carbohydrates, nucleic acids and lipoproteins, results in activation mediated by pattern recognition receptors (PRRs) found on the cell surface as well as on endosomal membranes (Hu et al. 1996; Muzio et al. 2000; Lee and Lee 2002; Block et al. 2007). Once these receptors are engaged by ligands (e.g., PAMPs), a cascade of cell signaling ensues which can result in a classical activation pathway, including the production and release of proinflammatory cytokines (e.g., tumor necrosis factor-α (TNF-α) and interleukin-1β (IL-1β), nitric oxide (NO) and superoxide. Alternatively, microglia can be activated to produce anti-inflammatory cytokines (e.g., arginase-1 and transforming growth factor-β), demonstrating the ability of these cells to regulate inflammation, allow for repair and promote homeostasis (Colton and Wilcock 2010). In addition to the typical PAMPs, sterile, non-pathogen related endogenous molecules associated with disease, called “danger/damage-associated molecular patterns” (DAMPs), are recognized by microglial PRRs (Halle et al. 2008; Chen and Nunez 2010; Duewell et al. 2010; Stewart et al. 2010; Beraud et al. 2011; Beraud and Maguire-Zeiss 2012). DAMPs also cause microglia to produce and release proinflammatory molecules. In fact, the ability of DAMPs to activate microglia has been tied to a number of neurodegenerative disorders, including Alzheimer’s disease, where fibrillar Aβ activates microglia leading to phagocytosis of the Aβ-containing plaque (Colton et al. 2000; Combs et al. 2001; El Khoury et al. 2003; Koenigsknecht and Landreth 2004; Jana et al. 2008). Likewise, α-synuclein, the PD protein prone to aggregation, directly activates microglia via a classical activation pathway and increases the expression of a subset of pattern recognition receptors (Zhang et al. 2005; Reynolds et al. 2008; Su et al. 2008; Theodore et al. 2008; Su et al. 2009; Lee et al. 2010a; Beraud et al. 2011; Beraud and Maguire-Zeiss 2012).

Importantly, there is also evidence for increased microglial activation in patients suffering from neurodegenerative diseases. Patients diagnosed with PD demonstrate an over six-fold increase in activated microglia compared to control patients when imaged using positron emission tomography (PET) and [^11^C](*R*)-PK11195, a ligand for the peripheral type benzodiazepine receptor (PBR) (Ouchi et al. 2005; Bartels and Leenders 2007; Ouchi et al. 2009; Bartels et al. 2010). These receptors are found on mitochondria in activated microglia and some neurons (Banati 2002) but ligand binding is increased when glia are activated. Interestingly, when PBR imaging was combined with PET for a dopamine transporter ligand ([^11^C]CFT), Ouchi et al. found an inverse correlation between midbrain [^11^C](*R*)-PK11195 and putamenal [^11^C]CFT and a positive correlation between midbrain [^11^C](*R*)-PK11195 and motor severity (Ouchi et al. 2009). As PD progressed the striatal dopamine transporter loss increased and the microglial activation encompassed nearly the entire brain (Ouchi et al. 2009). Although these in vivo imaging studies do not directly demonstrate that microglia are activated (e.g., additional highly specific microglial ligands are needed to confirm the PBR imaging data) they do support the idea that glial activation is ongoing in PD patients. Further evidence for an ongoing inflammatory state in PD comes from studies demonstrating increased levels of proinflammatory molecules (e.g., interleukins, TNF-α, interferon gamma) in patient blood and cerebrospinal fluid (CSF) as well as enhanced numbers of activated CD11b-positive microglia in post-mortem PD brains at autopsy compared with neurologically normal controls (McGeer et al. 1988; Barcia et al. 2004; El-Agnaf et al. 2006; Bartels and Leenders 2007; Brodacki et al. 2008; Bartels et al. 2010; Shi et al. 2011). Taken together, these studies suggest that persistent inflammation and microglial activation are fundamental characteristics of PD although the precise initiator, downstream consequences and nature of this activation remain to be fully determined.

### α-Synuclein, microglial activation and oxidative stress

As mentioned above mounting evidence implicates misfolded α-synuclein in the initiation and progression of microglial activation. For example, in transgenic mouse model where expression is limited to tyrosine hydroxylase containing neurons, overexpression of α-synuclein leads to increased numbers of activated microglia in the SNpc in the absence of dopamine neuron death (Su et al. 2008). Other α-synuclein transgenic models also support a role for this protein in neuronal dysfunction and degeneration, increased oxidative stress and microglial activation (Feany and Bender 2000; He et al. 2001; Dawson et al. 2002; Theodore et al. 2008; Kim et al. 2011a; Chesselet et al. 2012; Lastres-Becker et al. 2012). In addition, non-human primate and rodent studies employing neurotoxicants that target the nigrostriatal pathway demonstrate increased numbers of activated microglia prior to neuron death (Czlonkowska et al. 1996; Kohutnicka et al. 1998; Cicchetti et al. 2002; Depino et al. 2003; Wu et al. 2005; Zhang et al. 2005; Gerhard et al. 2006; Kim and Joh 2006; Liu 2006; Qian et al. 2006b; Sawada et al. 2006). Importantly, as mentioned above α-synuclein has a *direct* effect on microglial activation in vitro resulting in an overall increase in proinflammatory molecules and oxidative stress (Zhang et al. 2005; Su et al. 2008; Theodore et al. 2008; Su et al. 2009; Lee et al. 2010a; Beraud et al. 2011; Beraud and Maguire-Zeiss 2012). Furthermore, α-synuclein overexpression in animals and cell culture models demonstrates that α-synuclein protein aggregates can cause oxidative stress and increased cell vulnerability (Hsu et al. 2000; Parihar et al. 2008, 2009; Feng et al. 2010).

In PD patients, as detailed above, blood and CSF have detectable levels of proinflammatory molecules. Likewise, human studies support that α-synuclein is present in human plasma (El-Agnaf et al. 2006) and CSF (Mollenhauer et al. 2010, 2011). Interestingly, Tokuda et al. showed that CSF from PD patients had decreased levels of total α-synuclein but increased amounts of α-synuclein oligomers (Tokuda et al. 2010). α-Synuclein was also detected in the blood and brains from subjects with *SNCA* triplication PD (Miller et al. 2004). The presence of α-synuclein in the CSF is not limited to PD patients and is found in samples from patients with other synucleinopathies like dementia with Lewy bodies and Alzheimer’s disease (Noguchi-Shinohara et al. 2009). In vitro studies confirm that α-synuclein is localized to cell membranes, released from cells including neurons (Lee et al. 2005; Su et al. 2008; Emmanouilidou et al. 2010a; Feng et al. 2010; Jang et al. 2010) and taken up by surrounding glia (Lee et al. 2010b). Taken together, human and cell model studies suggest that α-synuclein is released from cells where it would be available for recognition by innate immune cells, promote activation and enhance oxidative stress.

Oxidative stress emanating from the cellular response to misfolded proteins, proteostatic dysfunction, is a common feature of neurodegenerative diseases and the PD-nigrostriatal pathway has increased levels of reactive oxygen species (ROS) and is particularly vulnerable to oxidative stress (Bossy-Wetzel et al. 2004; Surmeier et al. 2010). One source of oxidative stress emanates from the SNpc dopamine neurons themselves during autonomous pacemaking (Guzman et al. 2010) but neuronal stress can also originate from decreased cellular antioxidant responses as well as increased oxidative stress from surrounding activated glia (reviewed in (Miller et al. 2009)). For example, we have demonstrated that α-synuclein-directed microglial activation increases the production of NO (Beraud et al. 2011), which could adversely affect the health of surrounding neurons.

The master regulator of the cellular antioxidant response is the transcription factor NF-E2-related factor 2 (Nrf2; reviewed in (Johnson et al. 2008)). Nrf2 regulates the expression of phase-II detoxification and antioxidant enzymes that share a common DNA binding enhancer sequence, the antioxidant response element (ARE), recognized by this transcription factor. Interestingly, Nrf2 is implicated in the modulation of the innate immune system, including microglial activation following exposure to the well-known SNpc dopamine neuron toxicant 1-methyl-4-phenyl-1,2,3,6-tetrahydropyridine (MPTP; (Rojo et al. 2010)). In this study, MPTP-treated Nrf2 knockout mice demonstrated more nigrostriatal pathology than wild-type mice including enhanced gliosis (e.g., increased expression of GFAP and F4/80) and increased expression of proinflammatory molecules (e.g., TNF-α) and iNOS, an indicator of oxidative stress (Rojo et al. 2010). In addition, the overexpression of Nrf2 in astrocytes was shown to be neuroprotective against oxidative insults (e.g., hydrogen peroxide, MPTP; (Johnson et al. 2008; Chen et al. 2009)). Relevant to misfolded protein diseases, α-synuclein overexpression in *Drosophila* leads to dopamine neuron death that is rescued by the upregulation of phase II detoxification enzymes involved in glutathione metabolism, supporting the hypothesis that Nrf2-regulated genes are important in the cellular response to misfolded α-synuclein (Trinh et al. 2008).

In the remainder of this paper we present data supporting the perception that α-synuclein induces oxidative stress and promotes microglial inflammation in a conformation-dependent manner. Our data also supports the idea that cells respond to α-synuclein-induced oxidative stress by engaging the Nrf2-antioxidant response system resulting in increased expression of phase-II detoxification enzymes. Finally we discuss the concept that initial microglial activation and enhanced expression of antioxidant enzymes are appropriate cell responses and critical to maintain homeostasis; however, prolonged microglial activation is harmful and promotes neuronal vulnerability and disease progression.

## Methods

### Chemicals and reagents

Dulbecco’s Modified Eagle Medium (DMEM) and Minimum Essential Medium (MEM) were obtained from Cellgro, (St. Louis, MO). Fetal bovine serum was purchased from Hyclone (Logan, UT). All other reagents for cell culture and general use, if not indicated, were obtained from Invitrogen (Carlsbad, CA) or Sigma-Aldrich (St. Louis, MO).

### Animal studies

Transgenic mice with anti-oxidant response element driven human placental alkaline phosphatase expression (ARE, a kind gift of Dr. Johnson; (Johnson et al. 2002)) as well as, a compound transgenic mouse strain carrying both the ARE and human α-synuclein overexpression under the tyrosine hydroxylase promoter (SARE), were utilized in the specified experiments. The SARE compound transgenics were produced by breeding SYN_WT_+/+ (Richfield et al. 2002; Su et al. 2008) with hemizygous ARE (Johnson et al. 2002) mice to obtain SYN_WT_+/+::ARE+/− compound transgenics designated SARE. All ARE mice were heterozygous for the AREhPLAP gene. Mice were genotyped as previously described (Johnson et al. 2002; Richfield et al. 2002). All mice used for the experiments described here were male. Animals were maintained and treated in accordance with the regulatory standards of the Animal Welfare Act and approved for use by the Georgetown University Animal Care and Use Committee.

#### Gene expression analysis

The gene expression of various targets was analyzed using quantitative real-time PCR (qRT-PCR). Briefly, substantia nigra and striata were microdissected from ARE and SARE mice at 1, 6, and 12 months of age (*n =* 6 mice/age/genotype). Tissues were pooled from two mice of the same age and genotype and RNA was isolated using TRIzol® reagent (Invitrogen, Carlsbad, CA) per manufacturer’s protocol (*n =* 3 samples/region/genotype/age). RNA quantity and quality were assessed via standard spectrophotometric methods and Agilent 2100 Bioanalyzer (Santa Clara, CA; Lombardi Comprehensive Cancer Center Genomics & Epigenomics Shared Resource, Georgetown University Medical Center) following RQ1 DNase treatment (Qiagen, Valencia, CA). First strand cDNA from the pooled samples was synthesized from input RNA using the High-Capacity cDNA Archive Kit (Applied Biosystems, Carlsbad, CA) per manufacturer’s protocol. Gene expression was quantified in triplicate per sample using preconfigured TaqMan® low density arrays on the ABI Prism 7900HT Sequence Detection System (Applied Biosystems). The following primer/probe sets were used: *18S* (Hs99999901_s1); *ABCC1* (Mm00456156_m1); *CAT* (Mm00437992_m1); *GCLC* (Mm00802655_m1); *GCLM* (Mm00514996_m1); *GPX1* (Mm00656767_g1); *GPX4* (Mm00515041_m1); *GSR* (Mm00833903_m1); *GSS* (Mm00515065_m1); *GSTA2* (Mm00833353_mH); *HMOX1* (Mm00516004_m1); *HMOX2* (Mm00468921_m1); *NOX1* (Mm00549170_m1); *NQO1* (Mm00500821_m1); *PRDX1* (Mm01621996_s1); and *TXNRD2* (Mm00496771_m1). Expression levels were determined using the ∆∆Ct method with 18S rRNA as an endogenous control and age-matched ARE mice as the comparator. Statistical significance was analyzed using one-way ANOVA on the ∆Ct values with post hoc Student’s *t*-test (*p* < 0.05).

#### Immunohistochemistry

At the appropriate age, mice were perfused with 4 % paraformaldehyde under anesthesia and brains were removed, subjected to graded sucrose and microtome sectioned. Forty micron thick free-floating sections were processed for α-synuclein and tyrosine hydroxylase (TH) immunohistochemistry (IHC). All incubations were at room temperature unless noted otherwise. Briefly, sections were placed into net wells and tissue washed with Tris-buffered saline (TBS; 3 × 10 min), permeabilized for 5 min in TBS + 0.1 % Triton-X-100 (TBST) and blocked for 1 h in TBST + 10 % normal goat serum. Sections were then incubated with anti-α-synuclein (Thermo Fisher Scientific, Fremont CA; MS-1572-PABX; Syn211; 1:1000) and anti-TH (Millipore Corp., Temecula CA; AB152; 1:2,000) antibodies or no primary antibody overnight at 4 °C. Sections were then washed in TBST (3 × 10 min), followed by incubation with the appropriate fluorescent secondary antibodies (Life Technologies Corp; Molecular Probes Inc, Eugene OR; Alexa Fluor® 594 goat-α-mouse IgG and Alexa Fluor® 488 goat-α-rabbit; 1:1000) for 2 h. Sections were subsequently washed in TBST as above and nuclei stained with 4’,6-Diamidino-2-Phenylindole, Dihydrochloride (DAPI; 0.1 μg/ml; Life Technologies Corp). Unbound DAPI was removed by incubation with TBS (3 × 10 min) and sections were then mounted onto slides using Citifluor AF1 aqueous mounting media (Ted Pella Inc, Redding CA). Images were acquired using a Zeiss AxioPlan 2 microscope and Zeiss AxioCam HRm digital camera with AxioVision LSM image acquisition software (Carl Zeiss, Thornwood, NY).

#### Human placental alkaline phosphatase activity

One-, 6-, and 12-month-old ARE and SARE mice (*n =* 4/genotype) were perfused as described above and brains were removed and post-fixed in 4 % PFA for 1 h at 4 °C. Fixed brains were then subjected to graded sucrose, cyrostat sectioned (30 μm) and stored at −80 °C until histochemical analysis. Human placental alkaline phosphatase activity (hPLAP) was identified following colorimetric detection. Briefly, sections were removed from −80 °C, placed in a drying oven for 30 min at 42 °C, immersed in prewarmed HEPES balanced salt solution (HBSS; Mediatech Inc., Herndon, VA) and incubated at 65 °C for 2 h followed by a 40 min incubation with buffer containing levamisole (100 mM Tris–HCl, 50 mM MgCl2, 100 mM NaCl, 0.1 % Tween 20 and 5 mM levamisole) to inactivate endogenous phosphatases. Sections were then incubated in 5-Bromo-4-Chloro-3'-Indolyphosphate p-Toluidine Salt (BCIP)/Nitro-Blue Tetrazolium Chloride (NBT) reaction buffer (100 mM Tris–HCl, 50 mM MgCl2, 100 mM NaCl, 0.1 % Tween 20, 5 mM levamisole, 50 mg/mL BCIP and 37.5 mg/mL NBT) for 24 h on a slow rotating shaker in the dark. The reaction was stopped by incubation in PBS. Sections were analyzed using National Institutes of Health Image J 1.38X (NIH, Bethesda, MD) software. First, sections were photographed using an Axiophot microscope under 2.5X magnification. Anatomical regions of interest (e.g., substantia nigra) were outlined and subjected to the following conditions: the image was converted to 8-bit, scale set to 245.75 pixels/mm global, measurements set to area and limit to threshold, subtract background selected with a rolling ball radius of 50 pixels, light background and disable smoothing selected and a threshold value set at 120. An internal macro via the Image J program was used to aid in consistency. Measurements were calculated so that total values representing the area of deep purple (BCIP/NBT) stain for each individual animal could be tallied. The values for all brain sections for each individual animal were summed and subsequently analyzed by ANOVA using Graph Pad Prism 5 software (Graphpad Software Inc., La Jolla, CA).

### Expression, purification and characterization of α-synuclein

α-Synuclein was bacterially expressed in *Escherichia coli* BL21 (DE3) and purified as previously described, followed by lyophilization and storage at −20 °C until use (Maguire-Zeiss et al. 2006). The bacterial α-synuclein expression vector was a kind gift from Dr. Giasson (Giasson et al. 1999). To obtain misfolded α-synuclein the lyophilized protein was resuspended by sonication at 20 Hz (2 × 10 s bursts with 10 s rest between bursts) and diluted to 1 mg/ml in buffer (10 mM Tris–HCl, pH 7.5, 1 mM EDTA, 20 mM NaCl) followed by incubation for 5 days at 33 °C-37 °C (SYN) with or without 3.5 mM dopamine prepared in water (SYN^DA^). Buffer was incubated in the same manner and used as the buffer control for all treatments (Buffer or Buffer^DA^). For some experiments α-synuclein was incubated for 5 days at 37 °C with constant rotation (1,000 rpm) followed by size separation into high molecular weight SYN species (>150 kDa) and low molecular weight SYN as follows: 300 μg of misfolded SYN was placed onto a 150 kDa molecular weight cut off concentrator (MWCO; Thermo Fisher Scientific) and centrifuged at 3,000 × g for 30 min. The concentrate (HMW SYN) was resuspended in 100 μL of buffer. The flow through was collected and placed onto a 20 kDa molecular weight cut off concentrator and centrifuged at 3,000 g for 30 min. This low molecular weight concentrate (LMW SYN) was resuspended in 100 μL of buffer. The concentration of all protein samples was assessed using a Bio-Rad *DC* protein assay. The endotoxin content of SYN and buffer samples was evaluated using an E-TOXATE test kit following the manufacturer’s instructions (Sigma-Aldrich). The detection limit of the kit was 0.13 Endotoxin Units (EU)/ml (10 EU = 1 ng).

#### Western blot analysis

One microgram of SYN was added to denaturing sample buffer (62.5 mM Tris, pH 6.8, 10 % (v/v) glycerol, 2 % (w/v) SDS, 5 % (v/v) β-mercaptoethanol, and 1 % (w/v) bromophenol blue), boiled for 90 s and subjected to polyacrylamide gradient (4–16 %) gel electrophoresis under denaturing conditions followed by transfer to polyvinylidene difluoride (PVDF) membranes (PerkinElmer, Waltham, MA). A subset of SYN samples indicated in the figure legends were subjected to non-denaturing polyacrylamide gel electrophoresis, transferred to nitrocellulose membranes and processed for western blot analysis. All membranes were blocked in TBST/NFDM (20 mM Tris–HCl pH 7.5, 150 mM NaCl, 0.1 % (v/v) Tween, 5 % (w/v) non-fat dry milk). Mouse anti-α-synuclein primary antibody (1:1000; BD Biosciences, San Jose, CA) was used to probe for SYN conformers. Immune complexes were visualized on film following incubation with HRP-conjugated goat anti-mouse 2° antibody (1:2000; Chemicon, Temecula, CA) using Super Signal West Pico Chemiluminescent Substrate (Thermo Scientific, Waltham, MA).

#### Atomic force microscopy

Freshly cleaved muscovite mica was incubated in a mixture of 1-(3-aminopropyl) silatrane (APS) solution for 30 min to prepare APS-mica. Manipulated α-synuclein or buffer samples were added to the APS-mica and allowed to adhere for 2 min, washed with de-ionized water and dried with nitrogen gas (Shlyakhtenko et al. 2000, 2003). The mica was attached to a metal disc with double-sided tape for imaging. Images were acquired in tapping mode, using silicon tapping mode probes and a Multimode SPM Nanoscope IIIa system (Veeco/Digital Instruments, Santa Barbara, CA). Nominal spring constants of 60 N/m and a resonant frequency of 245 Hz were used.

### Cell culture

#### BV2 and primary microglia treatment

BV2 cells, a murine microglial cell line, were plated at a density of 5 × 10^5^ cells per well (6-well plates) in DMEM supplemented with 5 % fetal bovine serum and allowed to adhere for 24 h (Blasi et al. 1990; Horvath et al. 2008; Henn et al. 2009). One hour prior to treatment, serum-containing media were replaced with serum-free DMEM. Cells were subsequently treated with an equal volume of SYN (50 nM f.c.), SYN^DA^ (50 nM f.c.), Buffer or Buffer^DA^ in DMEM for 24 h. Following treatment, media were collected, centrifuged at 1,000 rpm for 2 min, placed in fresh tubes and stored at −20 °C until assayed. All treatments were preformed in triplicate on three separate biological replicates.

Primary microglia cultures were prepared from P1-P3 mouse cortices as previously described (Su et al. 2008), except that microglia were further isolated from mixed glial cultures (~DIV 14) by shaking at 125 rpm for 5 h at 37 °C on a rotary shaker with collection of the microglia-enriched medium (Beraud et al. 2011). Microglia were derived from C57/Bl6 or ARE mice (Johnson et al. 2002), plated at a density of 4 × 10^4^ cells per well (24-well plates on glass coverslips) in 0.5 mL of MEM supplemented with 0.01 % pyruvate, 0.6 % glucose and 5 % fetal bovine serum (microglia growth media) and allowed to adhere for 24 h. Cells were subsequently treated with 50 nM SYN or buffer control in microglia growth media for 24 h as described above. All treatments were preformed in triplicate on three separate biological replicates. Animals were maintained and treated in accordance with the regulatory standards of the Animal Welfare Act and approved for use by the Georgetown University Animal Care and Use Committee.

#### TNF-α secretion

TNF-α levels in the media of treated cells were measured by an enzyme-linked immunosorbent assay (ELISA) according to the manufacturer’s instructions (R&D Systems, Minneapolis, MN). All measurements were performed in triplicate on three separate biological replicates.

#### Nitric oxide release

Nitric oxide (NO) release into the media of treated cells was determined by measuring the stable NO metabolite, nitrite, using a Greiss reagent assay kit according to the manufacturer’s instructions (Invitrogen). All measurements were performed in triplicate on three separate biological replicates.

#### Human placental alkaline phosphatase activity in primary microglia

Primary microglia from ARE transgenic mice were cultured as described above and treated with 50 nM of SYN or buffer control for 24 h. Following treatment, cells were fixed with 4 % paraformaldehyde (w/v)/4 % sucrose (w/v) solution and stained for alkaline phosphatase activity using a BCIP/NBT kit according to the manufacturer’s instructions (Vector labs, Burlingame, CA). Resting, activated and phagocytic microglia displaying phosphatase activity (purple) as well as those displaying a nuclear counterstain (pink) were enumerated. Cells from nine random 20x ICC sections per sample from three coverslips were counted using a Zeiss AxioPlan 2 microscope (Ontario, NY). Four distinct groups of cells were counted based on staining and morphology (circularity, elongation and thickness of processes). Cells were considered inactivated/resting if they had thin and elongated morphology. Activated cells with thickened processes were categorized based on retraction and widening of processes. Cells were considered activated and phagocytic if they had a circular, ameboid shape. Cells lacking phosphatase activity were classified as unstained.

#### RNA extraction and qRT-PCR

Following treatment, RNA was harvested from cultured cells using an RNeasy mini kit and on-column DNase I digestion according to the manufacturer’s instructions (Qiagen, Valencia, CA). RNA purity was assessed using an Agilent 2100 Bioanalyzer and concentration measured using a NanoDrop 1000 spectrophotometer (Thermo Fisher Scientific). RNA was reverse transcribed in a 20 μl reaction volume using a High-Capacity cDNA Archive Kit (Applied Biosystems, Carlsbad, CA). The quality of the cDNA was verified following RT-PCR for β-actin expression. cDNA samples (10 μl) were then added to 90 μl of TaqMan® Universal PCR master mix and loaded onto TaqMan® Low Density Arrays (TLDA) preloaded with probes and primers for various targets and one endogenous control (Applied Biosystem; ABI Prism 7900HT Sequence Detection System; see Figure Legends and Tables). The results were analyzed using the relative quantification ΔΔCt method, normalizing samples to 18S rRNA, followed by normalization to the appropriate buffer treated controls. All measurements were performed in triplicate on three separate biological replicates. (Primers/Probes used: *TNF*α Mm00443258_m1, *IL1β* Mm0434228_m1, *NFĸB1* Mm0047361_m1, *TLR1* Mm0120884_m1, *TLR2* Mm00442346_m1, *TLR3* Mm00446577_g1, *TLR4* Mm00445273_m1, *TLR6* Mm01208943_s1, *TLR7* Mm00446590_m1, *TLR9* Mm00446193, *MYD88* Mm01351743 and *18S rRNA* Hs99999901_s1. For antioxidant response gene primers/probes see 2.2.1. Gene Expression Analysis. Statistical analysis was performed using a 1-way ANOVA followed by a Bonferroni post-hoc analysis on ∆Ct values with the significance level set at *P* ≤ 0.05.

#### Western blot analysis

BV2 and primary microglia cells were plated and treated as described above. Following treatment, cells were washed with ice-cold PBS and lysed on ice in modified RIPA buffer (50 mM Tris HCl pH 7.4, 1 % (v/v) NP-40, 0.25 % (w/v) sodium deoxycholate, 150 mM NaCl) supplemented with Protease Inhibitor Cocktail (for mammalian cells; Sigma-Aldrich). Cell lysates were subjected to gentle rotation for 20 min at 4 °C and then sonicated at 18 Hz (3 × 10 s bursts with 30 s rest between bursts). Lysates were cleared by centrifugation at 17,000 rpm for 10 min at 4 °C. Cleared lysates (25 μg) were treated as described above and subjected to denaturing polyacrylamide gel electrophoresis followed by transfer to PVDF membranes (PerkinElmer). Membranes were blocked in TBST/NFDM (20 mM Tris–HCl pH 7.5, 150 mM NaCl, 0.1 % (v/v) Tween, 5 % (w/v) non-fat dry milk) followed by incubation with mouse anti-heme oxygenase-1 primary antibody (HO-1; 1:1000; Abcam, Cambridge, MA). Immune complexes were visualized on film following incubation with HRP-conjugated goat anti-mouse 2° antibody (1:2000; Chemicon) using Super Signal West Pico Chemiluminescent Substrate (Thermo Scientific). Membranes were reprobed with α-tubulin (1:1000; Abcam), which served as the loading control. Densitometric analysis of digitized images was performed using the EC3 Imaging System (UVP, Upland, CA).

#### Iba-1 Immunocytochemistry

Microglia were plated on glass as described above and subsequently processed for immunocytochemistry (ICC). Following treatment, cells were washed with 1X PBS for 5 min, fixed in 4 % PFA at room temperature for 20 min, permeablized in 1X PBS containing 0.1 % triton X-100 for 5 min and blocked for 1 h with 1X PBS containing 10 % goat serum. Cells were incubated overnight at 4 °C with rabbit anti-Iba1 antibody (1:750; Wako, Richmond, VA) in blocking buffer. Antibody:antigen complexes were visualized following incubation with Alexa Fluor 594 conjugated goat anti-rabbit IgG secondary antibody 1:500. Unbound 2° antibody was removed by washing with 1X PBS containing 0.1 % triton X-100. Cells were counterstained with DAPI (1:5000) in 1X PBS for 5 min and following two washes with 1X PBS the cover glasses were mounted in Citifluor (Ted Pella, Redding, CA), sealed and imaged.

### Statistical analyses

All statistical analyses were carried out using Graphpad Prism 5 (Graphpad Software Inc., La Jolla, CA) or JMP 9.0.0 (SAS Institute, Inc., Cary, NC). ANOVA were performed, followed by Bonferroni’s *post hoc* test or Student’s *t*-test where appropriate. All data are reported as means ± standard deviation. *P*-values ≤ 0.05 were considered significant.

## Results

### Increased expression of antioxidant enzymes in mice that overexpress human α-synuclein

**Fig. 1:**
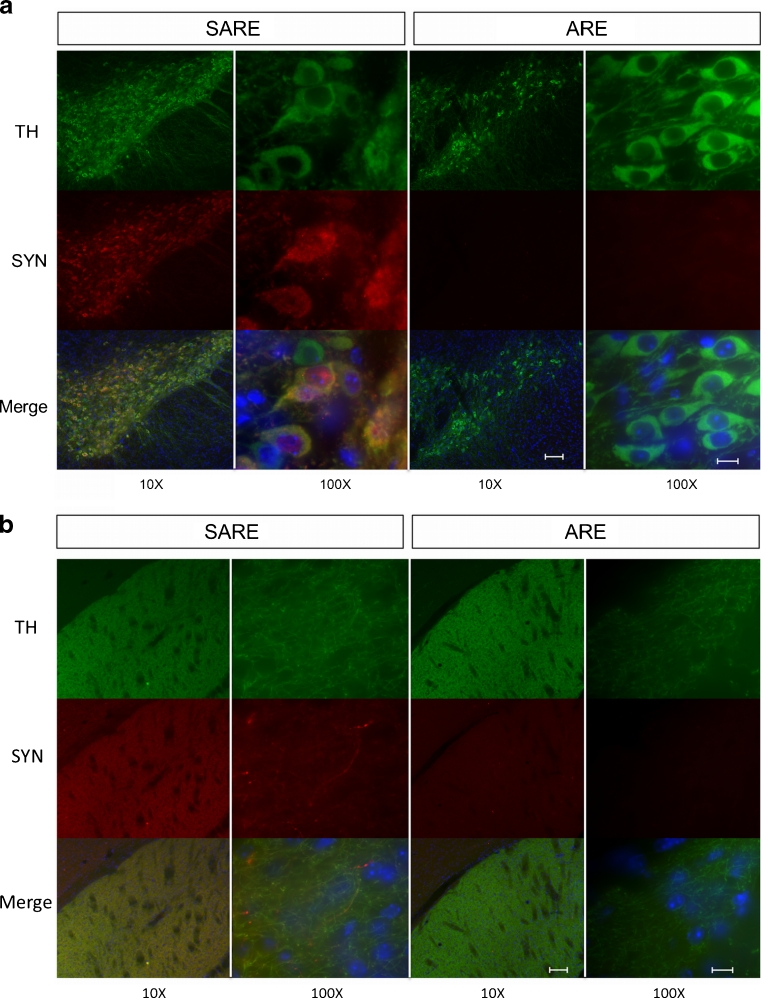
α-Synuclein and TH co-localize in the SNpc and striatum of SARE mice. Substantia nigra **a** and striatal **b** images from 1-month old SARE and ARE mice following co-immunohistochemistry for tyrosine hydroxylase (TH; *green*) and α-synuclein (SYN; *red*) demonstrating co-localization (Merge; *yellow*) of these proteins in mice that overexpress human α-synuclein under the control of a rat TH promoter (SARE). Images were taken at 10X (scale bar = 100 μm) and 100X (scale bar = 10 μm) magnifications

**Fig. 2:**
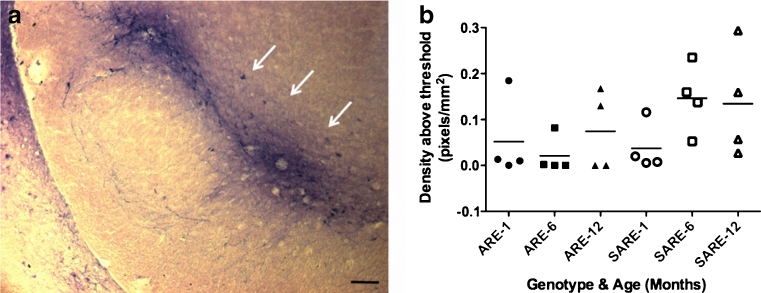
Antioxidant responses in vivo*.* The SNpc of 1-, 6- and 12-month old ARE and SARE mice (*n =* 4/genotype/age) were processed for hPLAP activity measurements using a BCIP/NBT staining protocol. **a** Image of phosphatase activity in the SNpc of an SARE mouse (12-months of age) demonstrating robust activity in this region of the brain (white arrows). Images were taken at 10X (scale bar = 100 μm) magnification. **b** The density of phosphatase activity was determined from the SNpc of BCIP/NBT stained tissue for all mice and reported as pixels/mm^2^. There is no statistically significant difference between α-synuclein overexpressing mice (SARE) and ARE mice in phosphatase activity

Using two existing transgenic mouse lines, we produced a new mouse, SARE, which overexpresses human α-synuclein under the direction of the TH promoter and is capable of reporting an antioxidant response via expression of human placental alkaline phosphatase (hPLAP). The promoter used to drive hPLAP expression in the ARE and SARE mice contains 51-base pairs of the rat NAD(P)H:quinone oxidoreductase 1 (NQO1) promoter including the core antioxidant response element (5’-GTGACnnnGC-3’)(Johnson et al. 2002). It was previously established by Johnson et al., that cultured neurons and glia from ARE mice express hPLAP when treated with tBHQ (tert-butylhydroquinone), a molecule known to activate ARE through an Nrf2-mediated mechanism (Johnson et al. 2002; Kraft et al. 2004). Therefore we reasoned that these reporter mice crossed with α-synuclein overexpressing mice would reveal spatial and temporal synuclein-initiated antioxidant responses. Towards this goal, groups of SARE and ARE mice at 1-, 6- and 12-months of age were prepared for IHC and hPLAP activity. As shown in Fig. 1, SARE mice exhibit co-localization of TH and α-synuclein at all ages in SNpc neurons and attendant presynaptic terminals in the striatum. There were no apparent pathological effects of α-synuclein overexpression at these ages following microscopic inspection. We next examined the ARE antioxidant response by colorimetric identification of hPLAP activity (Fig. 2a). Here we demonstrate that the SNpc exhibits an increased antioxidant response compared with surrounding brain regions. Using this method we did not detect a statistically significant difference in phosphatase activity between the ARE and SARE mice (Fig. 2b).

**Fig. 3:**
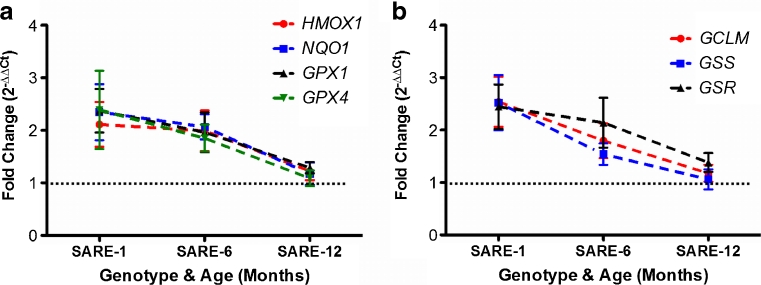
α-Synuclein overexpression increases the expression of antioxidant genes in the striatum. Quantitative RT-PCR was performed on cDNA obtained from 1-, 6- and 12-month old ARE and SARE mouse striata. **a** SARE mice had significantly higher expression of genes associated with the detoxification of hydrogen peroxide and quinones (*HMOX1, NQO1, GPX1* and *GPX4*) at 1- and 6-months of age compared with age-matched ARE mice. **b** SARE mice had significantly higher expression of genes associated with glutathione metabolism (*GCLM, GSS* and *GSR*) at 1- and 6-months of age compared with age-matched ARE mice. Expression values were normalized to 18S rRNA as an internal control. All SARE measurements at 1- and 6-months of age were significantly different than ARE at the same ages (*P* < 0.05). The dashed line represents the gene expression level of ARE mice. Data expressed as fold change (2^-∆∆ct^) ± S.D

We next employed a more sensitive assay, qRT-PCR, to determine whether a subset of known Nrf2-regulated genes displayed altered expression when α-synuclein was overexpressed. RNA was prepared from the SN and striata of 1-, 6- and 12-month old SARE and ARE mice, reverse transcribed and gene expression levels quantified using preconfigured TaqMan® low density arrays. Using 1-month ARE mice as the comparator, α-synuclein overexpression increased the nigral and striatal gene expression level for a number of the antioxidant enzymes interrogated at 1-month of age (Table 1). In the SN of SARE mice, genes that regulate glutathione metabolism: glutamate-cysteine ligase (*GCLC*), glutamate-cysteine ligase regulatory subunit (*GCLM*) and glutathione synthetase (*GSS*) were increased nearly 2-fold compared to ARE mice. Genes associated with the detoxification of hydrogen peroxide and quinones were also upregulated in 1-month old SARE mice (glutathione peroxidase-1/*GPX1*, glutathione peroxidase-4/*GPX4*, heme oxygenase-1/*HMOX1,* NAD(P)H dehydrogenase, quinone 1/*NQO1;* catalase/*CAT*). *ABCC1*, which encodes for a superfamily of ATP-binding cassette transporters, including the multidrug resistance-associated protein-1, and functions with oxidized glutathione to transport molecules across membranes was increased 2-fold in 1-month old SARE mice. Likewise gene expression levels in the striatum were upregulated in 1-month old SARE mice. In the SN the expression levels for these genes returned to ARE levels at subsequent timepoints. Interestingly, expression levels at 1- and 6-months of age for SARE striatal genes associated with the detoxification of hydrogen peroxide and quinones as well as glutathione metabolism remained upregulated compared with age-matched ARE mice (Fig. 3).

**Table 1:** Oxidative stress gene expression changes in 1-month old α-synuclein overexpressing mice

Entrez Gene ID	Common Name	Description	Fold change in gene expression by brain region^1^	
			Substantia Nigra	Striatum
17250	ABCC1	ATP binding cassette C	↑ 2.0	↔
12359	CAT	Catalase	↑ 2.0	↑1.4
14629	GCLC	Glutamate-cysteine ligase-catalytic	↑ 1.8	↔
14630	GCLM	Glutamate-cysteine ligase-modifier	↑ 2.0	↑ 2.5
14775	GPX1	Glutathione peroxidase 1	↑ 1.5	↑ 2.4
14854	GSS	Glutathione synthetase	↑ 1.8	↑ 2.5
14782	GSR	Glutathione reductase	↑ 1.8	↑ 2.5
625249	GPX4	Glutathione peroxidase 4	↑ 1.5	↑ 2.2
15368	HMOX1	Heme oxygenase 1	↑ 1.6	↑ 2.1
237038	NOX1	NADPH oxidase I	ND	↔
18104	NQO1	NADPH dehydrogenase quinone 1	↑ 1.6	↑ 2.5
18477	PRDX1	Peroxiredoxin 1	↔	↑ 2.3
26462	TXNRD2	Thioredoxin reductase 2	↔	↑ 2.4

Expression data in each brain region represents changes in 1-month old mice that overexpress human α-synuclein and carry the AREhPLAP transgene (SARE) compared to AREhPLAP mice (ARE), which do not overexpress human α-synuclein. The expression levels of GSTA2 and HMOX2 were below the limit of detection and are not included in this table. ^1^ND indicates expression levels below the limit of detection; ↔ indicates no significant difference in gene expression between SARE and ARE mice. *P* ≤ 0.05

### Microglia respond differently to specific types of α-synuclein

**Fig. 4:**
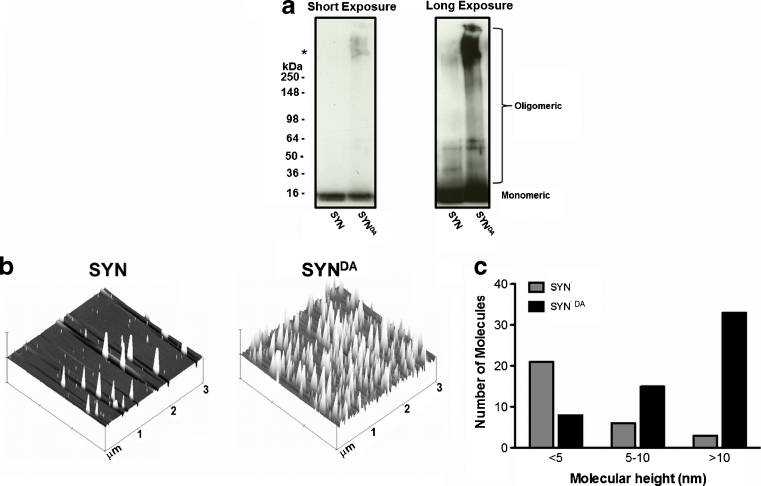
Characterization of misfolded α-synuclein. α-Synuclein was incubated at 33–37 °C in the absence (SYN) or presence of dopamine (SYN^DA^) and characterized by western blot analysis following polyacrylamide gel electrophoresis under denaturing conditions (SDS-PAGE) or by AFM under native conditions. Dopamine modification of α-synuclein caused an increase in high molecular weight aggregates. **a** Western blot analysis of manipulated α-synuclein demonstrated an increase in SDS-stable, high molecular weight oligomers following dopamine modification (SYN^DA^). α-Synuclein samples were subjected to 4–16 % SDS-PAGE and immunoblotted for α-synuclein. Both short and long film exposures are shown; *denotes the stacking/resolving gel interface. Short exposure corresponds to a 2 s exposure time for the film, while the long exposure is a 15 s exposure time. **b** AFM demonstrated an increase in α-synuclein aggregates following incubation with heat and dopamine. **c** Quantification of AFM molecular height images demonstrated that incubation of α-synuclein in the presence of dopamine (SYN^DA^; *black*) resulted in a 10-fold increase in aggregates >10 nm with a concomitant decrease in molecules <5 nm in height compared to α-synuclein without dopamine (SYN; *grey*). Molecular height images are shown at 3 μm × 3 μm × 3 μm

We have previously demonstrated that α-synuclein containing β-sheet structures increases microglial activation that is consistent with a classical activation pathway (Beraud et al. 2011). Here we extend those studies and ask whether different forms of misfolded α-synuclein similarly activate microglia. As discussed above, α-synuclein will readily misfold into oligomers and high molecular weight aggregates in response to molecular crowding, agitation, temperature, and following incubation with dopamine (DA). Therefore we expressed and purified recombinant human wild-type α-synuclein and induced aggregation using temperature, rotation and/or DA (SYN; SYN^DA^). Buffer controls (i.e., no α-synuclein) were similarly treated (Buffer, Buffer^DA^). The resultant α-synuclein conformers and buffer controls were subsequently tested for endotoxin contamination, which was found to be below the detectable limit of the assay (< 0.013 ng/ml). To determine whether SDS-stable oligomers were present in these samples, SYN and SYN^DA^ were subjected to polyacrylamide gel electrophoresis under denaturing conditions followed by western blot analysis for α-synuclein. As shown in Fig. 4a, both SYN and SYN^DA^ contain monomeric as well as SDS-stable oligomers of α-synuclein. However, SYN^DA^ was comprised of significantly more high molecular weight α-synuclein aggregates than SYN. We also characterized SYN and SYN^DA^ under nondenaturing conditions using atomic force microscopy (AFM) to visualize and quantify the height distribution of the manipulated proteins (Fig. 4b & c). The predominant molecular height of SYN was less than 5 nm (70 %), representing monomeric α-synuclein, while approximately 10 % of the α-synuclein molecules were aggregates of greater than 10 nm. Conversely, dopamine-modified α-synuclein contained a significantly greater number of aggregates with a molecular height >10 nm (59 %) and nearly 1.9-fold more detectable molecules at this height than SYN. Together, these data show that both SYN and SYN^DA^ contain monomer and aggregates of α-synuclein and that dopamine-modification enhances the propensity for α-synuclein to form larger aggregates.

**Fig. 5:**
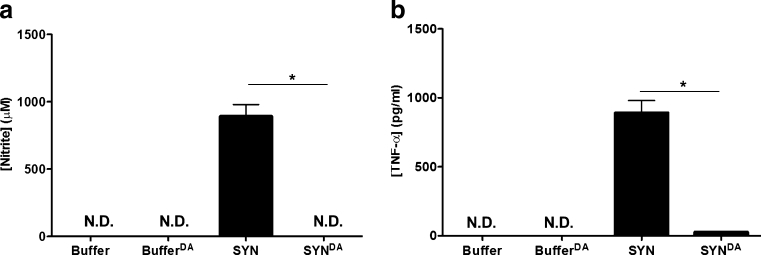
SYN induces a classical activation pattern in BV2 cells. SYN causes conformer-specific increases in proinflammatory molecule expression and release. **a** BV2 cells were treated with 50 nM SYN, 50 nM SYN^DA^ or equal volumes of the appropriate buffer control for 24 h. Following treatment, a Greiss reagent assay was performed on the conditioned media to determine nitrite production. Conditioned media from SYN-treated BV2 cells had a substantial amount of nitrite (70 μM); in contrast there was no detectable (N.D.) NO in the media of cells treated with SYN^DA^ or in media from buffer treated cells; (*p < 0.05). **b** BV2 cells were treated as described above and TNF-α protein was quantified in the conditioned media by ELISA. TNF-α release from BV2 cells was significantly increased in SYN-treated BV2 cells compared to the DA-modified SYN-treated (SYN^DA^) cells; there was no detectable (N.D.) TNF-α in media from buffer treated cells; (*p < 0.05). All values represent three biological replicates with treatments in triplicate

To determine whether these differentially modified forms of α-synuclein induce a proinflammatory response in microglia we exposed BV2 cells, an immortalized mouse microglial cell line, to SYN and SYN^DA^ as well as buffer controls and assayed for prototypical proinflammatory molecules. Conditioned media from treated BV2s were assayed for the amount of nitrite, a stable NO metabolite. NO secretion from cells treated with SYN was significantly increased compared with buffer- or dopamine-modified α-synuclein (SYN^DA^; Fig. 5a; *P* ≤ 0.05). In fact, NO was undetectable following SYN^DA^, Buffer or Buffer^DA^ treatment of BV2 cells. We next quantified the amount of a common proinflammatory cytokine, TNF-α, released by the treated BV2s. Similar to the NO production, exposure to SYN induced the release of ~900 pg/ml of TNF-α, which is typical of classically activated microglia while SYN^DA^- or buffer-treatment had little effect on TNF-α release (Fig. 5b; *P* ≤ 0.05; (Beraud et al. 2011)). Likewise, *TNF*α gene expression was upregulated 14-fold in BV2s treated with SYN, while SYN^DA^-treated BV2s exhibited no significant difference in expression compared to buffer-treated cells (Table 2).

**Table 2:** Inflammatory gene expression changes following SYN treatment of BV-2 microglia

Entrez Gene ID	Common Name	Description	Fold change in gene expression following treatment^1^	
			SYN	SYN^DA^
21897	TLR1	Toll-like receptor 1	↔	↔
24088	TLR2	Toll-like receptor 2	↑ 2.0	↔
142980	TLR3	Toll-like receptor 3*	↑ 3.7	↔
21898	TLR4	Toll-like receptor 4	↓ 0.7	↔
21899	TLR6	Toll-like receptor 6	↔	↔
170743	TLR7	Toll-like receptor 7*	↓ 3.0	↔
81897	TLR9	Toll-like receptor 9	↔	↔
20778	CD36	Scavenger receptor class b member 1	↔	↔
16176	IL1β	Interleukin 1 beta*	↑ 56	↑ 2.7
21926	TNFα	Tumor necrosis factor alpha*	↑ 14	↔

BV-2 microglia were treated with 50 nM of SYN, SYN^DA^ or appropriate buffer for 24 h. ^1^ ↔indicates no significant difference in gene expression compared to buffer treated cells *P* ≤ 0.05; ND indicates expression levels below the limit of detection. *denotes a significant difference in gene expression fold change between SYN and SYN^DA^ treated cells at *P* ≤ 0.05

Since we have previously shown that *IL1β* mRNA expression is robustly increased in BV2 cells following α-synuclein treatment (Beraud et al. 2011), we next determined whether α-synuclein treatment altered the expression of this cytokine. When compared to their respective buffer controls, *IL1β* gene expression was significantly increased following exposure of BV2 cells to either SYN or SYN^DA^. However, the fold-increase in *IL1β* gene expression was 56-fold for the SYN-treated BV2 cells and only 2.7-fold for BV2s treated with SYN^DA^ (Table 2). Taken together, these data establish that a mixture of α-synuclein containing both monomeric and high molecular weight conformers of this protein significantly augments the expression, production and release of classical proinflammatory molecules from BV2 cells, while α-synuclein misfolded in the presence of dopamine (SYN^DA^) causes a dampened response, despite the presence of high molecular weight α-synuclein species suggesting that the structure of α-synuclein modified by dopamine is different than α-synuclein modified in the absence of this neurotransmitter.

Microglial activation can be mediated by engagement of DAMPs to cognate pattern recognition receptors and these interactions are structure dependent. Therefore, we next asked whether the expression of a subset of these receptors was altered in BV2 cells following α-synuclein treatment (Table 2). Specifically, we interrogated genes encoding toll-like receptor (*TLR*) *1*, *2*, *3*, *4*, *6*, *7* and *9*, scavenger receptor *CD36* as well as proteins involved in TLR and microglial activation (NFĸB; MyD88). SYN-treated BV2 cells demonstrated increased expression of *TLR2* and *TLR3* with a down regulation of *TLR4* and *TLR7* compared to buffer-treated BV2s. There were no significant changes in the expression levels for *TLR1, 6, 9*, *CD36, NFĸB or MYD88*. In contrast, BV2s exposed to dopamine-modified α-synuclein (SYN^DA^) did not exhibit a significant gene expression change for *TLRs*, *CD36, NFkB or MYD88* (Table 2), which supports our previous data demonstrating that SYN^DA^ did not induce a proinflammatory response.

**Fig. 6:**
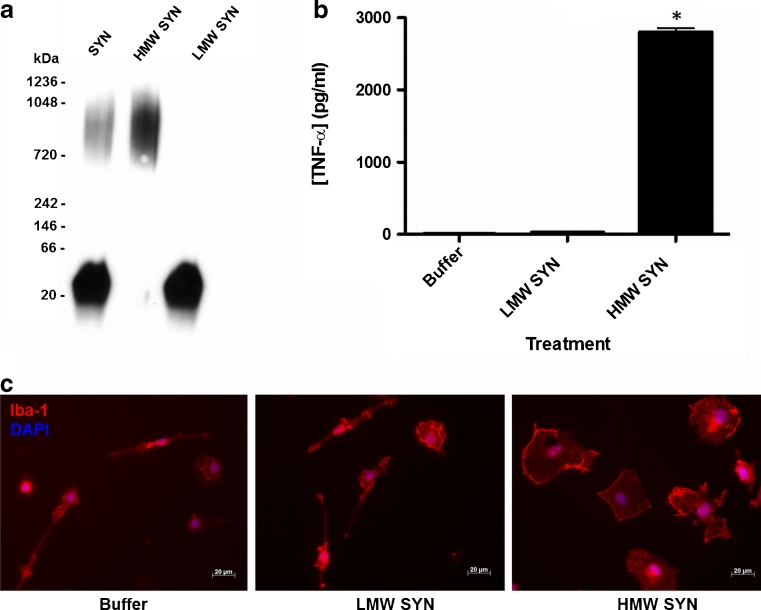
HMW α-synuclein activates microglia. a Representative α-synuclein western blots of misfolded α-synuclein under native conditions. Purified human recombinant α-synuclein was incubated at 37 °C with 1000 rpm rotation for 5 days to induce misfolding (SYN). Misfolded SYN was then separated into high molecular weight (HMW SYN) and low molecular weight (LMW SYN) fractions using MWCO concentrators. **b** Primary microglia were treated with buffer, LMW SYN (50 nM) or HMW SYN (50 nM) for 24 h. Following treatment, the conditioned media was evaluated for TNF-α protein secretion using an ELISA. Cells treated with HMW SYN released significantly more TNF-α than Buffer or LMW SYN treated microglia (*P < 0.05, *n =* 3). **c** Primary microglia were treated with buffer, 50 nM of LMW SYN or 50 nM HMW SYN for 24 h. Cells were fixed and immunostained for the microglial marker Iba-1 (*red*) followed by a DAPI nuclear counterstain (*blue*). The majority of primary microglia treated with buffer or LMW SYN display prototypical ramified morphology of resting microglia while glia treated with HMW SYN display the characteristic ameboid shape of activated microglia (scale bar = 20 μm)

Since SYN which contains both monomeric and oligomeric protein caused an increase in TNF-α, IL-1β and several TLRs and dopamine-modified α-synuclein had either no or a less robust effect on these molecules we reasoned that protein structure is important for glial activation. To address this hypothesis, we misfolded α-synuclein and enriched for high (> 150 kDa; HMW) and low molecular weight (~ 20 kDa; LMW) structures using molecular weight cut off concentrators. Fig. 6a shows the relative purity of these fractions following western blot analysis of non-denatured α-synuclein before (SYN) and after separation (HMW SYN; LMW SYN). We then exposed primary microglial cultures derived from mouse cortices to HMW SYN, LMW SYN or buffer and quantified TNF-α release (Fig. 6b). Only the HMW SYN fraction robustly increased the release of this prototypical proinflammatory cytokine. In addition, morphological analysis of Iba1+ cells revealed a predominant increase in ameboid microglia following treatment with HMW SYN, a structure consistent with highly activated phagocytic microglia (Fig. 6c). These results are consistent with the hypothesis that α-synuclein structure directs the extent and the nature of the microglial response.

### α-Synuclein induces an antioxidant response in microglia

**Fig. 7:**
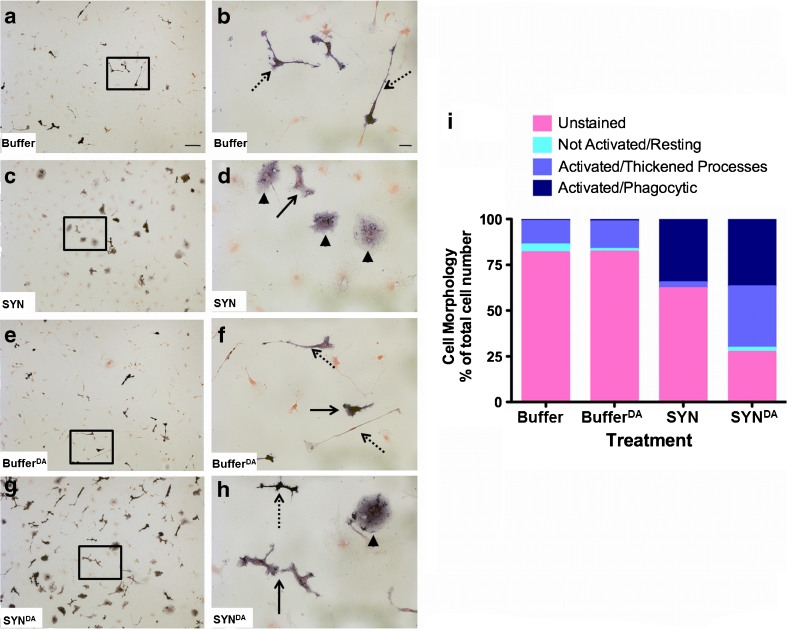
Exposure of primary microglia to α-synuclein increases antioxidant expression. Primary microglia from ARE transgenic mice were histochemically stained with BCIP/NBT (*purple*) to detect hPLAP activity and nuclear red counterstained (*pink*). Microglia exposed to Buffer (**a**, **b**) or Buffer^DA^ (**e**, **f**) displayed less phosphatase activity than SYN-treated cells and also exhibited the prototypic morphology of resting microglia (dashed arrows; **a**, **b**, **e**, **f** & **i**) with a few glia that were activated but not phagocytic (solid arrows). In contrast, cells exposed to SYN or SYN^DA^ displayed increased numbers of microglia with the characteristic amoeboid morphology of phagocytic microglia compared to the other exposure paradigms (solid arrowheads; **c**, **d**, **g**, **h** & **i**). Interestingly, microglia exposed to SYN^DA^ had the greatest percentage of microglia expressing phosphatase activity with nearly 75 % of the treated microglia activated, as demonstrated by thickened processes and ameboid shape (solid arrow and solid arrowhead, respectively; **g**, **h** & **i**). Scale bar for 10x (**a**, **c**, **e**, **g**) and 40x (**b**, **d**, **f**, **h**) images represent 100 µm and 20 µm respectively. Boxes in **a**, **c**, **e**, and g denote the area for 40x images **b**, **d**, **f** and **h**. Cell counts were performed on nine random 20x images from each sample and categorized based on staining and morphology as outlined in Materials and Methods (**i**)

As discussed above one consequence of NO and proinflammatory molecule production is an overall increase in oxidative stress and microglia respond by increasing the expression of antioxidant response genes in an attempt to maintain homeostasis (Chowdhury et al. 2009; Bast et al. 2010). Since the upregulation of these phase-II detoxification enzymes regulated in part by the transcription factor, Nrf2, provides cellular protection from oxidative stress, we next asked whether exposure of microglia to various forms of α-synuclein enabled an antioxidant response. Primary microglia derived from ARE reporter mice (Johnson et al. 2002, 2008) were exposed to SYN, SYN^DA^ or buffer controls and analyzed for hPLAP activity (Fig. 7). All treated cells had a subset of glia with phosphatase activity; however, the majority of microglia (>75 %) exposed to buffer only maintained a ramified morphology with small cell bodies and long processes typical of non-activated microglia (Panels a, b, e & f; dashed arrows and quantified in Panel i). However, these microglia also had a subset of cells with larger cell bodies and shorter processes, consistent with an activated but not phagocytic morphology (Panel f; solid arrow and quantified in Panel i). In contrast, ARE microglia exposed to SYN or SYN^DA^ displayed a robust change in morphology to an ameboid shape, consistent with microglia that are highly activated and/or phagocytic (Panels c, d, g & h; solid arrowheads and quantified in Panel i). The primary microglia exposed to dopamine-modified α-synuclein (SYN^DA^) had increased numbers of microglia with thickened processes (activated but not phagocytic) compared to the SYN-treated microglia.

We next investigated whether the antioxidant genes that were upregulated in the α-synuclein overexpressing mice (Table 1) were altered in microglia exposed to misfolded α-synuclein. The overall response following SYN or SYN^DA^ exposure of BV2s was an upregulation of antioxidant response gene expression (Table 3), which is in alignment with the phosphatase activity measurements in primary microglia (Fig. 6). There was also a robust increase in *NADPH oxidase 1* (*NOX1*) gene expression in SYN-treated microglia (240-fold increase). This enzyme generates superoxide ion promoting the formation of NO and IL-1β in LPS-treated microglia (Cheret et al. 2008) and its upregulation here is in line with our data demonstrating that SYN-treated microglia have an activation profile that includes increased levels of NO, TNF-α and IL-1β. For nearly all of the interrogated genes the response to SYN was significantly different from SYN^DA^-treated BV2 cells, translating into a less robust antioxidant response following SYN^DA^ treatment as well as a weakened proinflammatory response. Importantly, there were no significant differences in gene expression levels when Buffer-treated BV2 cells were compared to Buffer^DA^-treated cells suggesting that the gene expression changes following SYN^DA^ treatment was due to SYN^DA^ not free DA.

**Table 3:** Oxidative stress gene expression changes following SYN treatment of BV-2 microglia

Entrez Gene ID	Common Name	Description	Fold change in gene expression following treatment^1^	
			SYN	SYN^DA^
14775	GPX1	Glutathione peroxidase 1*	↓ 0.7	↔
18477	PRDX1	Peroxiredoxin 1*	↑ 3.5	↑1.4
14858	GSTA2	Glutathione S transferase, alpha 2*	↑ 500	↑ 60
18104	NQO1	NADPH dehydrogenase quinone 1*	↑ 6.0	↑ 4.0
15368	HMOX1	Heme oxygenase 1*	↑ 4.0	↑ 2.0
15369	HMOX2	Heme oxygenase 2	ND	ND
12359	CAT	Catalase*	↑ 2.4	↑ 2.0
14630	GCLM	Glutamate-cysteine ligase-modifier	↑ 3.0	↑2.5
14629	GCLC	Glutamate-cysteine ligase-catalytic	↔	↔
14854	GSS	Glutathione synthetase*	↓ 0.6	↓ 0.9
14782	GSR	Glutathione reductase*	↑ 2.5	↑ 1.5
17250	ABCC1	ATP binding cassette C*	↑ 2	↑ 1.3
237038	NOX	NADPH oxidase 1	↑ 240	↑ 61
26462	TXNRD2	Thioredoxin reductase 2*	↓ 0.4	↓ 0.6
625249	GPX4	Glutathione peroxidase 4*	↓0.7	↔

BV-2 microglia were treated with 50 nM of SYN, SYN^DA^ or appropriate buffer for 24 h. ^1^↔ indicates no significant difference in gene expression compared to buffer treated cells *P* ≤ 0.05; ND indicates expression levels below the limit of detection. *denotes a significant difference in gene expression fold change between SYN and SYN^DA^ treated cells at *P* ≤ 0.05

**Fig. 8:**
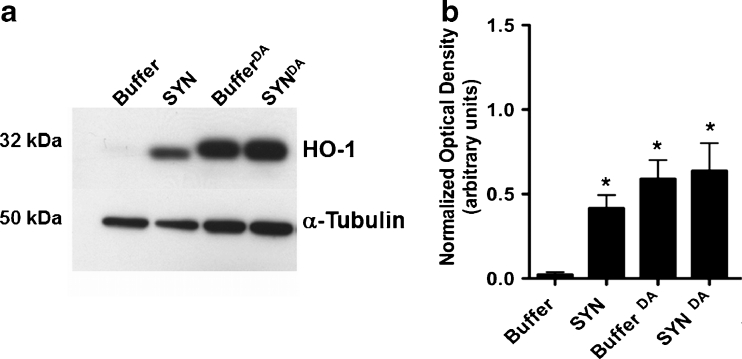
Exposure of microglia to α-synuclein or dopamine increases HO-1 protein expression. **a** Representative HO-1 western blot analysis of BV2 lysates. BV2 cells were treated with 50 nM of SYN or SYN^DA^ or equal volumes of the appropriate buffer control for 24-h. Protein lysates were prepared and subjected to 10 % SDS-PAGE and immunoblotted for HO-1 (~32 kDa). Blots were re-probed for α-tubulin (~50 kDa) as a loading control. **b** Immunocomplexes were quantified by densitometric analysis. The HO-1 signal was normalized to the loading control. Cells treated with SYN or SYN^DA^ had significantly higher levels of HO-1 expression compared to buffer alone (^*^ *p* < 0.05, 1-way ANOVA followed by Student’s *t* post-test). Additionally, the presence of DA alone was enough to significantly increase HO-1 expression. Cells treated with buffer that was incubated in the presence of DA (Buffer^DA^) had significantly higher levels of HO-1 than buffer incubated in the absence of DA (*p < 0.05, 1-way ANOVA followed by Student’s *t* post-test). Values represent three biological replicates with treatments in triplicate

**Fig. 9:**
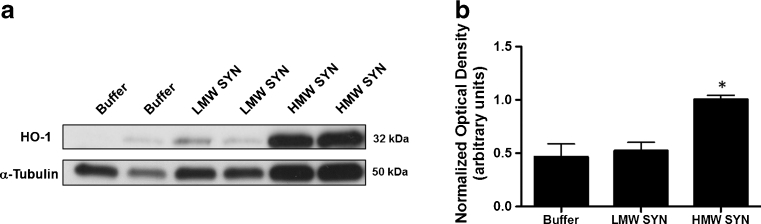
Exposure of microglia to HMW α-synuclein increases HO-1 protein expression. **a** Representative HO-1 western blot analysis. Primary microglia were treated with 5 nM of LMW SYN, HMW SYN or buffer control for 24-h. Protein lysates were prepared and subjected to 4–20 % SDS-PAGE and immunoblotted for HO-1 (~32 kDa). Blots were re-probed for α-tubulin (~50 kDa) as a loading control. **b** Immunocomplexes were quantified by densitometric analysis. The HO-1 signal was normalized to the loading control. Cells treated with HMW SYN had significantly higher levels of HO-1 expression compared to buffer or LMW SYN (*p < 0.05, 1-way ANOVA followed by Student’s *t post-test)*

Since, the antioxidant enzyme, HO-1 is upregulated following exposure to α-synuclein and in our α-synuclein overexpressing mice (Table 1; Fig. 3 and (Kitamura et al. 1998a, b; Tanaka et al. 2006; Bast et al. 2010; Beraud et al. 2011; Lastres-Becker et al. 2012)), we next examined HO-1 protein levels from microglia treated with different forms of α-synuclein. Both SYN and SYN^DA^ caused a significant upregulation of HO-1 protein (Fig. 8a & b; *P* ≤ 0.05). Buffer^DA^ treatment also increased HO-1 expression supporting previous evidence that DA alone promotes oxidative stress and increases HO-1 expression (Schmidt et al. 1999). HMW SYN, which caused an increase in TNF-α and a robust morphological change in primary microglia also increased HO-1 expression in these cells while LMW SYN did not affect the expression of this antioxidant (Fig. 9). Taken together, these data demonstrate a robust antioxidant response following treatment of microglia with high molecular weight aggregates of α-synuclein as demonstrated by an increase in ARE-directed hPLAP activity, morphological changes typical of phagocytic and activated microglia and enhanced expression of proinflammatory molecules and antioxidant response enzymes. In contrast, dopamine modified α-synuclein did not induce a robust TNF-α response nor increase NO but did increase the expression of HO-1. HO-1 protein was also upregulated in microglia exposed to DA alone in the absence of any proinflammation.

## Discussion

In 1988, the potential role for inflammation in Parkinson’s disease (PD) pathogenesis was suggested when McGeer’s group demonstrated an increase in reactive microglia (HLA-DR-positive) in the substantia nigra of PD brains (McGeer et al. 1988). Since that time, a GWA study identified an HLA locus (rs3129882 of HLA-DR) as a risk factor for sporadic PD (Hamza et al. 2010). Furthermore, in vivo imaging supports that glial activation is present in the early stages of PD and this activation increases as the disease progresses (Ouchi et al. 2005; Gerhard et al. 2006; Ouchi et al. 2009). Genetic and neurotoxicant animal models targeting the nigrostriatal pathway also substantiate a role for microglial activation and oxidative stress in response to PD-relevant stressors prior to neuron death (Czlonkowska et al. 1996; Cicchetti et al. 2002; Depino et al. 2003; Kim and Joh 2006; Liu 2006; Qian et al. 2006a; Su et al. 2008, 2009). However, the exact role activated microglia play in PD is unclear as microglia are associated with both repair and toxicity. Furthermore the term “activation” does not adequately describe the complex morphological and functional change microglia undergo when reacting to changes in the microenvironment (discussed in (Colton and Wilcock 2010; Harry and Kraft 2012)).

Conventional dogma classifies activated microglia as those glia with larger cell bodies and thicker, less-ramified processes and that release proinflammatory cytokines such as TNF-α and IL-1β, as well as molecules indicative of increased oxidative stress (e.g., NO). Additional subclasses within the classical activation categorization have been proposed more recently based on the expression of cytoactive factors that result in inflammotoxic-, excitotoxic- and redox-mediated activation states, which further supports the idea that complex and intricate molecular interactions result from microglial activation (Colton and Wilcock 2010). Microglia are also activated in an alternative pathway that promotes immunological resolution via the production of neurotrophic factors, anti-inflammatory cytokines (i.e., IL-10, IL-4), arginase I, matrix metalloproteinases and antioxidant response proteins (i.e., HO-1; reviewed in (Colton and Wilcock 2010)). Therefore, microglia can quickly react and respond to danger/damage signals in a complex manner that facilitates the destruction and/or removal of these molecules as well as the initiation of subsequent repair and/or immunological resolution. This ability places microglia in a position to significantly affect brain homeostasis.

**Fig. 10:**
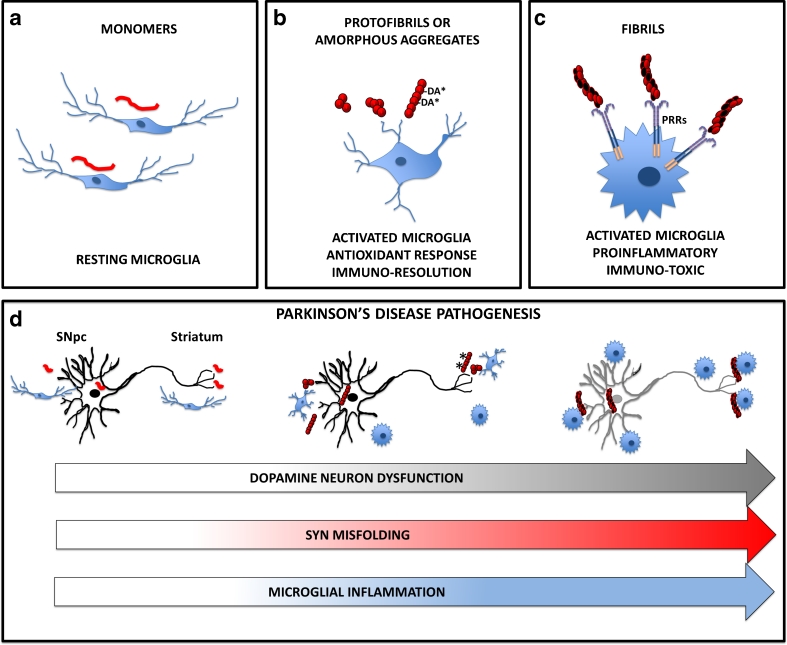
Schematic diagram depicting the effect of α-synuclein on microglia and PD. α-Synuclein promotes microglial activation in a structure dependent manner contributing to PD pathogenesis. Data presented in this paper supports the hypothesis that a specific structure is required to directly activate microglia and that antioxidant responses are in response to α-synuclein overexpression and direct glial activation. **a** Monomeric α-synuclein (red line) does not directly activate microglia. **b** Protofibrils stabilized by dopamine (−DA*) and amorphous aggregates of α-synuclein increase microglial expression of antioxidant enzymes and have an attenuated proinflammatory response (modest increase in *IL1β*), which we hypothesize leads to immuno-resolution rather than toxic inflammation. **c** Fibrils of α-synuclein directly activate microglia in a classic proinflammatory pathway (high levels of NO, *NOX1*, TNF-α and *IL1β*). Even though antioxidant enzymes are upregulated they cannot quell this robust glial activation. We further suggest that fibrillar α-synuclein is recognized by specific microglial PRRs, which facilitate a proinflammatory pathway. These highly activated cells would be immune-toxic for surrounding neurons. **d** Parkinson’s disease pathogenesis schematic. SNpc dopamine neurons normally express α-synuclein and this protein is enriched in striatal presynaptic dopamine terminals. If α-synuclein maintains a random coil structure this protein does not activate microglia (far left). When α-synuclein forms a protofibillar structure (e.g., due to overexpression, oxidative stress, the presence of dopamine quinone) microglia respond by increasing the expression of antioxidant enzymes and take on an activated morphology that we suggest is an attempt to return the microenvironment to homeostasis (middle). However, as opposed to pure microglial cultures, in this in vivo setting there is neuronal-glial crosstalk with factors released from the stressed dopamine neurons available to signal local glia causing a mild inflammatory response that is not due to a direct glial-synuclein interaction (middle). As α-synuclein continues to misfold into fibrils (far right and *red arrow*) and oxidative stress is enhanced in the local microenvironment, more microglia become activated in a robust proinflammatory pathway (*blue arrow*) leading to increased dopamine neuron dysfunction (*gray arrow*). Similar to Panel (c.), at this point the antioxidant response cannot suppress the ongoing glial activation

In neurodegenerative disorders, including PD, it is hypothesized that persistent oxidative stress and proinflammatory activation of microglia contribute to the death of surrounding neurons (e.g., toxicity); however, it is also possible that activation of microglia is an attempt at immunological resolution and that only with continued neurodegeneration is the balance tipped toward toxicity (Fig. 10). We suggest the following critical factors determine the outcome of microglial activation (e.g., toxicity vs. resolution): the microenvironment (e.g., nigrostriatal system), the temporal aspects of the microglial response (e.g., early vs. late in disease) and the inciting danger/damage molecular pattern (e.g., specific conformation of α-synuclein). In the studies presented here we focused on α-synuclein as the danger/damage molecular pattern and characterized the antioxidant response in an animal model of overexpression as well as the proinflammatory and antioxidant responses in microglia.

Our data support the hypothesis that α-synuclein contributes to oxidative stress through a pathway that induces complex microglial activation as well as antioxidant responses and in addition requires a specific protein structure (Fig. 10). First, we demonstrate that in an animal model of α-synuclein overexpression there is an increase in the nigrostriatal expression of genes involved in cellular responses to oxidative stress. We interrogated genes that are known to be regulated by the master regulator of antioxidant responses, Nrf2, in a novel animal model of α-synuclein overexpression in the context of an antioxidant reporter transgene (SARE) generated by crossing mice homozygous for human α-synuclein (SWT+/+; (Su et al. 2008)) with AREhPLAP transgenic mice (Johnson et al. 2002). Relevant to the studies presented here, at one-month of age α-synuclein transgenic mice (SWT+/+) exhibit increased numbers of activated microglia in the SNpc as well as increased expression of *TNF*α in the SN and striatum, demonstrating an early microglial activation driven by the overexpression of α-synuclein (Su et al. 2008). Here we also show that genes associated with the detoxification of hydrogen peroxide and quinones as well as those involved in glutathione metabolism remain increased in the striatum of α-synuclein overexpressors (SARE mice) compared with age-matched controls (ARE mice) up to 6-months of age. More studies are needed to define the importance of these gene expression changes but it is interesting to speculate that the upregulation of these genes is in response to the early microglial activation previously described and that these early antioxidant responses are capable of restoring homeostasis since this model does not display significant nigrostriatal pathology or glial activation at older ages (Richfield et al. 2002). Other models of α-synuclein overexpression also display glial activation supporting the ability of this protein to induce inflammation in vivo (Theodore et al. 2008; Chesselet et al. 2012). However, the cellular response to oxidative stress is complex, partially mediated through engagement of the Nrf2 transcription factor to its cognate DNA binding site (Johnson et al. 2008) but other transcription factors including activator protein 1 (AP1), cAMP response element binding protein (CREB), activating transcription factor 2 (ATF2) and nuclear factor kappa B (NFĸB) activator are also important for the regulation of these genes (Kim et al. 2011b). Adding to the complexity some genes (*NQO1*, glutathione S-transferase P, [*GSTP1*] and methallothionein [*MT1 & 2*]) that contain a typical Nrf2 antioxidant response element (ARE) are also regulated by another Cap‘n’Collar transcription factor, Nrf1 (Leung et al. 2003; Ohtsuji et al. 2008). Although mechanistic studies are needed to confirm that Nrf2-mediated responses are at play when α-synuclein is overexpressed in our model, Lastres-Becker et al. used adeno-associated viral vector delivery of human α-synuclein in Nrf2 knockout (Nrf2-KO) mice and demonstrated increased nigrostriatal pathology (Lastres-Becker et al. 2012). Furthermore, Nrf2-KO mice exposed to the nigrostriatal neurotoxicant, MPTP, have enhanced microgliosis with increased levels of proinflammatory markers and decreased expression of proteins associated with alternative activation, supporting a role for Nrf2 in modulating microglial activation states (Rojo et al. 2010).

Second, here we confirm and extend previous work demonstrating that α-synuclein directly activates microglia in a classical proinflammatory activation pathway. We previously demonstrated that misfolded human α-synuclein directly activates microglia via the classical pathway with increased expression of a subset of pattern recognition receptors, known to bind DAMPs (Beraud et al. 2011). In the current study we asked whether different types of misfolded α-synuclein induce the same microglial activation pattern and in addition promote an antioxidant response. Our data confirms a highly complex microglial response to different types of α-synuclein. We show that α-synuclein containing high molecular weight SDS-stable species activates microglia in a classical proinflammatory activation pathway. In contrast, ‘monomeric’ α-synuclein does not induce a proinflammatory response. However, high molecular weight aggregation as defined by gel electrophoresis or AFM is not the only criteria for activation since in our hands α-synuclein modified by dopamine (SYN^DA^) does not cause a robust proinflammatory response. This suggests that a specific secondary structure is required for activation that is present in SYN but not SYN^DA^. Our data also support this idea since the expression of TLR pattern recognition receptors is only increased in the species of α-synuclein that leads to proinflammation (i.e., SYN not SYN^DA^). Mechanistic studies are underway to test this hypothesis.

Third, we show that along with a robust classic proinflammatory activation of microglia by α-synuclein there is also evidence that these glia are oxidatively stressed as indicated by an increased production of NO and expression of *NOX1*. Again the strength of these responses appears to be dependent on α-synuclein structure/modification as *GPX1, PRDX1, GSTA2, NQO1, HMOX1, GSS, GSR* and *ABCC1* expression changes were all increased in misfolded SYN-treated cells above the SYN^DA^-treated glia. For example, SYN^DA^-treated microglia only increased the expression of *glutathione-S-transferase* (*GSTA*) 60-fold while the response to misfolded SYN was a 500-fold increase in gene expression. However, we did observe that SYN^DA^ altered microglial morphology and ARE-driven hPLAP activity and increased the expression of Nrf2-directed antioxidant enzymes. Relevant to our observations, when microglia derived from Nrf2-KO mice were exposed to α-synuclein, the glia were unable to increase the expression of two antioxidant enzymes (HO-1 and NQO1) and they took on a more proinflammatory profile (Lastres-Becker et al. 2012) demonstrating that Nrf2 can regulate microglial responses to DAMPs. We also characterized the HO-1 response in microglia since it is upregulated in response to α-synuclein and dopamine [Figs. 8 & 9 and (Schmidt et al. 1999; Beraud et al. 2011; Lastres-Becker et al. 2012)]. In addition to Nrf2 (Lastres-Becker et al. 2012), HO-1 expression is regulated by other transcription factors, including AP1, NFĸB, hypoxia-inducible factor 1 (HIF1) and stress response proteins (Schipper et al. 2009) leading to a complex spatial and temporal pattern of expression and function (Kraft et al. 2006, 2007; Chen et al. 2009; Rojo et al. 2010; Granado et al. 2011; Koh et al. 2011; Lastres-Becker et al. 2012). For example, HO-1 can be neuroprotective, promoting intracellular degradation of soluble α-synuclein (Song et al. 2009) and defending against hydrogen peroxide oxidative damage as well as β-amyloid toxicity (Le et al. 1999; Wang et al. 2012). However, HO-1 is also able to facilitate bioenergetic failure and in a toxicant model this leads to dopamine neuron injury (Lee et al. 2006; Schipper et al. 2009). With regard to PD, HO-1 is found surrounding the hallmark proteinaceous PD inclusion, the Lewy body, and is increased in PD brains where its function is still unclear (Schipper et al. 1998). Here we show that *HMOX1* expression is increased when human α-synuclein is overexpressed in vivo and in cultured microglia exposed to SYN and SYN^DA^. On the other hand, HO-1 protein levels were increased and not substantially different from each other when BV2 cells were treated with SYN, SYN^DA^ or Buffer^DA^. It appears that the increase in HO-1 following SYN-treatment is in response to oxidative stress emanating from glial activation by misfolded α-synuclein but following SYN^DA^ or Buffer^DA^ increased HO-1 is a glial response to oxidized dopamine. In both scenarios this robust HO-1 response might be an attempt to promote homeostasis, which is effective when microglia are not yet fully activated (i.e., Buffer^DA^ & SYN^DA^) but fails when glia are driven to a robust classical activation state (i.e., misfolded SYN). Further studies are necessary to determine the exact functional state of these microglia. As such we are continuing to investigate the exact structure of these misfolded α-synuclein conformers, which should facilitate the development of molecular tweezers (Prabhudesai et al. 2012) that could target specific α-synuclein structures.

The other interesting target for a novel PD therapy is the antioxidant transcription factor, Nrf2. PD is a progressive disorder and increased age is the greatest risk factor for developing this disease. In a mouse model of spontaneous accelerated aging, Nrf2 expression and translocation to the nucleus was decreased compared with normally aging mice (Tomobe et al. 2012), suggesting that an age-dependent decline in transcriptionally active Nrf2 could add to the risk of developing diseases related to oxidative stress such as PD. As mentioned above studies using Nrf2/KO mice demonstrated that the loss of this transcription factor makes neurons more vulnerable to oxidative stress-related toxicants. Since Nrf2 upregulates the expression of many antioxidant genes altering the activity of this transcription factor would affect a wide variety of antioxidant systems, which may represent a stronger approach than targeting only one antioxidant enzyme (reviewed in (Hybertson et al. 2011)). Nrf2 activators can be derived from plants for example, sulforaphanes are from cruciferous vegetables and curcumin is derived from the spice, turmeric. Mixtures of natural compounds have also been formulated to produce low dose synergistic activation of Nrf2 (e.g., Protandim; LifeVantage Corp; (Hybertson et al. 2011)). Synthetically produced activators are also available such as tBHQ, synthetic triterpenoids, and dimethylfumarate (BG-12; Biogen Idec). These Nrf2 activators are effective in both in vitro and in vivo models of cancer, diabetes, inflammatory disorders and neurodegenerative diseases (Calabrese et al. 2010; Hu et al. 2010; Kundu and Surh 2010; Kwak and Kensler 2010; Hybertson et al. 2011; Negi et al. 2011; Perumal and Khan 2012). In addition many of these compounds (e.g., sulforaphanes, BG-12 and curcumin) are being tested in human clinical trials for a number of immune-related and neurologic conditions including multiple sclerosis and Alzheimer’s disease ((Hybertson et al. 2011; Perumal and Khan 2012); www.ClinicalTrials.gov). Since the regulation of Nrf2 activity is dependent on the ability of this protein to disengage from KEAP1 and translocate to the nucleus and there is evidence of cross talk between this antioxidant response pathway and others (e.g., adipogenic, ubiquitin, inflammatory) the development of small molecules that mimic Nrf2 activity and/or weaken its interaction with KEAP1 also represent a fertile area of pharmacologic exploration (Wakabayashi et al. 2010).

In conclusion, we have reviewed the literature, which supports the idea that microglial activation is important in PD progression and have shown that the PD-related protein, α-synuclein, incites an increase in antioxidant response enzyme expression in vivo and in cultured microglia. Furthermore, we demonstrated that the specific structure of misfolded α-synuclein dictates the glial activation pattern and antioxidant responses. The development of small molecules that either prevent the formation of or disrupt the structure of the proinflammatory form of α-synuclein in combination with natural or synthetic Nrf2 activators will enhance the armamentarium to combat PD.
